# A modified single-armed microsurgical vasoepididymostomy for epididymal obstructive azoospermia: intraoperative choice and postoperative consideration

**DOI:** 10.1186/s12894-020-00692-5

**Published:** 2020-08-12

**Authors:** Nachuan Liu, Peng Li, Erlei Zhi, Chencheng Yao, Chao Yang, Liangyu Zhao, Ruhui Tian, Huixing Chen, Yuhua Huang, Yuexin Yu, Zheng Li

**Affiliations:** 1 Department of Andrology, the Center for Men’s Health, Urologic Medical Center, Shanghai Key Laboratory of Reproductive Medicine, Shanghai General Hospital, Shanghai Jiao Tong University, Shanghai, 200080 China; 2 Anhui Medical University, Hefei, 230032 China

**Keywords:** Obstructive azoospermia, Male infertility, Vasoepididymostomy, Patency, Pregnancy

## Abstract

**Background:**

To evaluate the clinical outcomes and the duration required for the sperm to return to the ejaculate after a modified single-armed 2-suture longitudinal intussusception vasoepididymostomy (SA-LIVE).

**Methods:**

From March 2015 to December 2018, 134 patients with epididymal obstruction azoospermia underwent the modified single-armed vasoepididymostomy at Shanghai General Hospital. The outcomes and clinical findings were documented and evaluated. The mean follow-up period was 17 (range: 3–36) months.

**Results:**

Patency was assessed by the return of sperm in the ejaculate. The overall patency rate was 55.2%, and the patency rates were 58.9, 40.7, 36.4, and 58.9% for bilateral surgery, unilateral surgery, proximal anastomosis, and distal anastomosis, respectively. The average time to achieve patency was 4.11 ± 2.74 months. In the first 6 months, 87.8% (65/74) patency patients reported sperm in the ejaculate. The overall pregnancy rate was 40.9% (29/66) at the follow-up of 3–36 months, and the natural pregnancy rate was 30.3% (20/66). The natural pregnancy rate was 32.1% post-bilateral surgery and 33.3% for the site of distal anastomosis; surprisingly, it was 0% for the site of proximal anastomosis.

**Conclusion:**

Modified SA-LIVE is safe and may achieve favorable patency and pregnancy rates. When double-armed sutures are not accessible, single-armed may be preferable. The expected patency time was within 1 year. Moreover, because of the low natural pregnancy rate for proximal anastomosis, sperm banking is preferred to SA-LIVE.

## Background

Azoospermia affects 1% of the general population and 10–15% of infertile men [1]. Obstructive azoospermia, primarily caused by epididymal obstruction, is diagnosed in approximately 40% of azoospermic men [2, 3]. Microsurgical vasoepididymostomy (MVE) has been established as a more cost-effective alternative for men with obstructive azoospermia than direct assisted reproductive techniques (ART) [4]. MVE necessitates superior surgical skills and meticulous surgical technique. Cornell et al. first reported that the single-armed 2-suture longitudinal intussusception vasoepididymostomy (SA-LIVE) was similar to that of the double-armed procedure in an animal study [5]. Zhao et al. reported the modified single-armed VE technique with favorable patency in a human study trial [6]. Previous studies have shown that a high patency rate may be related to factors, such as epididymal fullness, unilateral or bilateral procedure, and site of anastomosis [7]. However, only a few studies have assessed the clinical outcomes and the time of sperm returning to the ejaculate after SA-LIVE. Thus, in the present study, we aimed to evaluate the fertility outcomes of MVE in 134 males with epididymal obstruction and identify the putative predictors for natural pregnancy. Also, novel intraoperative procedures and decision-making post-operation were explored.

## Methods

### Subjects and laboratory examination

From March 2015 to December 2018, 158 patients who suffered from epididymal obstruction azoospermia (EOA) underwent VE surgery in our center (four patients were misdiagnosed with NOA previously, and one patient had VE in another hospital 2 years ago). However, during the follow-up, we collected the data from the semen analysis of 134 patients from our center or telephonic follow-up. In this cohort, 80/134 (55.9%) of the patients had a previous history of urological or genital infection before the study, and the etiology of the other 54 cases remained unknown. None of the patients underwent a vasectomy in this study.

All patients underwent semen analysis at least three times before surgery. No sperm was detected in a centrifugal (1500×*g*) semen assay, and ejaculate fructose tests were positive. The sex hormone levels, such as follicle-stimulating hormone (FSH) and testosterone (T), in the serum, were within normal limits. Scrotal ultrasonography showed dilation of the epididymal tube if present. These were objective measurements for obstructive azoospermia [3].

### Diagnostic clinical condition and criteria

The diagnostic criteria for inclusion in the study were as described previously [8]: obstruction was suspected when the infertile male had normal ejaculate volume with azoospermia; the physical examination showed non-atrophic testes with normal vas deferens bilaterally, slightly swollen epididymis, and bilateral or unilateral hard epididymal nodules; normal serum total T and FSH levels; the ultrasonography showed the dilation of epididymal tube without dilation of the ejaculatory duct or seminal vesicle. The patients with chromosomal or sex chromosomal abnormalities, history of vasectomy, or whose female partners were reported to be infertile, were excluded from the current study.

### Surgical procedure

#### General preparation

All patients underwent scrotal exploration under general anesthesia. Surgeries were performed by two experienced surgeons. Testis was exposed and biopsy was performed as follows: a touch-prep is made by blotting the cut surface of the testis several times on a glass slide and adding a drop of human tubal fluid and a coverslip. The samples were examined under high power using a light microscope with phase contrast that revealed the presence of sperm with tails and facilitated the assessment of motility. Subsequently, normal spermatogenesis was confirmed [9, 10]. All patients provided written informed consent before the study.

#### Microsurgical preparation

A 24-gauge angiocatheter sheath was used to cannulate the lumen of the vas deferens, and Trypan blue was injected to judge the patency of the seminal vesicle side. A sufficient length was freed to allow the most proximal convoluted portion of the vas to be later brought to the lateral aspect against the epididymis without tension.

#### Microsurgical SA-LIVE approach

**Fig. 1:**
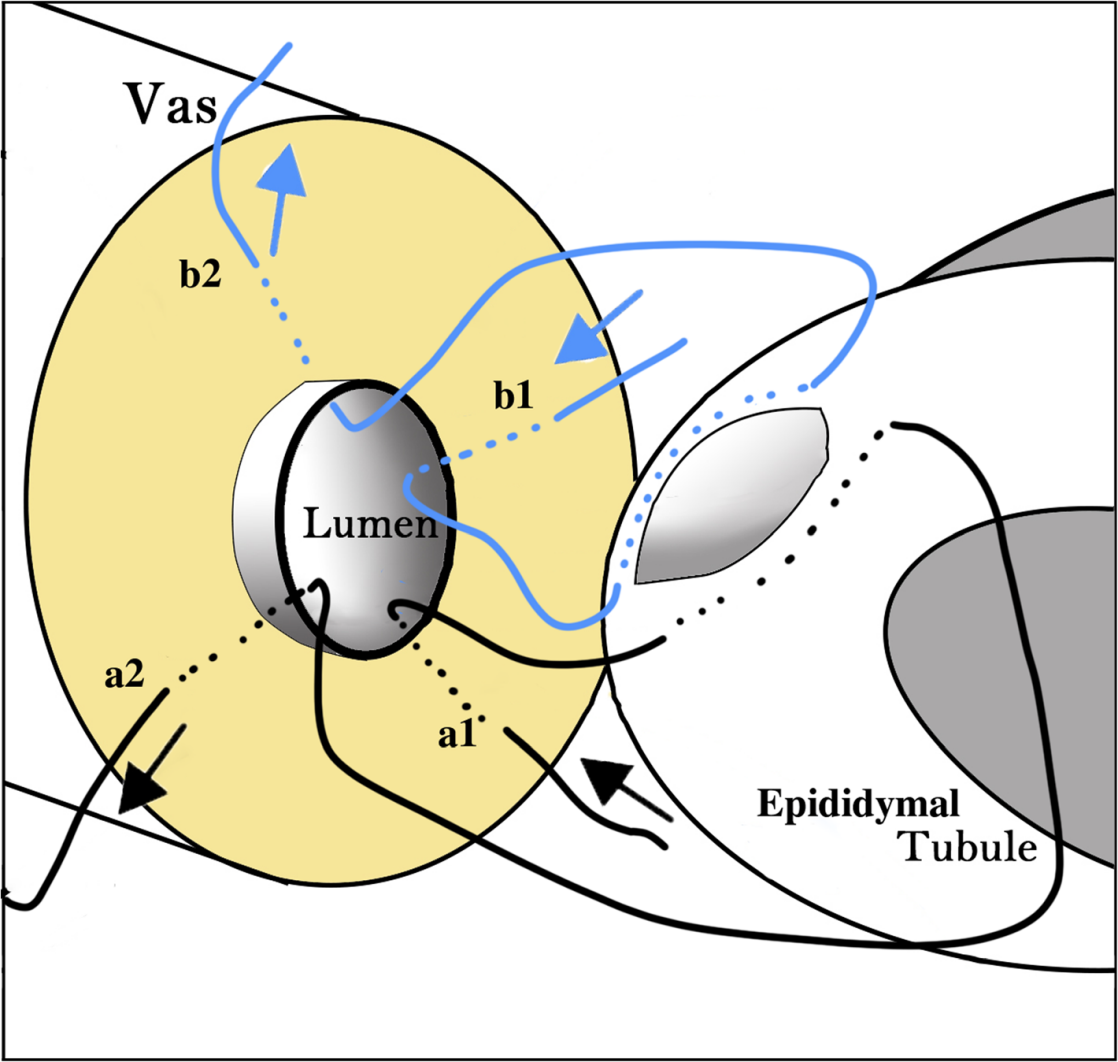
Placement of sutures in modified SA-LIVE. The needles were sequentially placed outside-in (a1 and b1) through the mucosal layer of the vas deferens, parallelly through the epididymal tubule, then placed inside-out (a2 and b2) through the mucosal layer of the vas deferens

A Carl Zeiss S88 operating microscope (Carl Zeiss Shanghai Co., Ltd., Shanghai, China) was used to perform the microsurgical procedure at a magnification of 8–15X. A dilative epididymal tubule was selected and dissected under microscopic guidance. Subsequently, anastomosis was performed using the modified SA-LIVE technique (Fig. 1). Two single-armed 10–0 nylon sutures (Ethicon W2790, length 200 mm, circle 3/8) were prepared for the intussusception sutures. The first suture was placed in an outside-in fashion through the mucosal layer of the vas deferens at point a1. Then, the needle was used to pierce the lateral aspect of the epididymal tubule and placed longitudinally. The second 10–0 single-armed proline suture was placed identically through point b1 on the vas deferens, parallel to the first suture on the contralateral side of the epididymal tubule. The two needles were used to parallelly pierce the epididymal tubule laterally within and longitudinally on the outside of the tubule. The epididymal tubule was opened longitudinally between the two sutures using a micro knife, and the exuded epididymal fluid was examined for sperm [10]. If the sperm or sperm fragments were present, the needles were pulled through the wall of the epididymis and placed in an inside-out manner through the full layer of the vas at positions a2 and b2. Finally, all the sutures were tied together (a1 to a2 and b1 to b2), and the epididymal tubule was gently intussuscepted into the lumen of the vas deferens. Then, the epididymal tunic was secured to the vassal muscle and adventitia with an 8–0 nylon suture. The epididymal and testicular sperms were cryopreserved during VE as a backup of the procedure.

### Postoperative management

Most of the patients were discharged home on the 1st day after the surgery. They were advised to refrain from any heavy lifting and sports activity for 8 weeks post-surgery, and asexual abstinence was instructed for 4 weeks. Semen analyses were initiated at 4 weeks after surgery, and every month after that, until pregnancy was achieved. Patency was defined as the sperm concentration > 1 million/ml in at least one postoperative ejaculate sample. Pregnancy was defined as the establishment of a fetal heartbeat. The follow-up information was obtained by clinic visits and telephonic contacts. Patients without a postoperative semen analysis or patients lost to follow-up were excluded from the report, and the follow-up time was at least 3 months.

### Statistical analysis

The patency and pregnancy rates of each group (unilateral and bilateral MVE group and the proximal and distal anastomosis group) were calculated. The chi-square test was used for all analyses. *P* < 0.05 indicated statistical significance.

## Results

From March 2015 to December 2018, a total of 158 patients diagnosed with an epididymal obstruction underwent modified SA-LIVE in our center; among these, 134 were followed up in a prospective study. Reportedly, 80 patients had a history of epididymitis or orchitis; full epididymis could be palpated easily with inflated nodes. The diagnostic ultrasonography findings also proved the existence of inflammation in the scrotum based on the thin net-like ectasia of the epididymal tube with the inner diameter up to 0.4 mm [11]. The mean age of the included patients was 32.1 ± 6.7 (range: 23–50) years, and the mean follow-up was for 17 ± 3.3 (range: 3–36) months. The mean FSH and T levels were 4.3 ± 2.4 mIU/mL and 5.0 ± 2.9 ng/mL, respectively. The mean testicular size was 15.4 ± 3.3 cm^3,^ as measured by ultrasonography (Table 1).

**Table 1:** Preoperative characteristics and intraoperative choices in all 134 patients

Items	Value
Age (year), mean	
Patients	32.1 ± 6.7 (ranged 23–50)
Female partners	27.2 ± 3.7 (ranged 20–43)
Serum FSH (mIU/mL), mean	4.3 ± 2.4
Serum total testosterone (ng/mL), mean	5.0 ± 2.9
Testicular size (cm^3^), mean	15.4 ± 3.3
Surgery, n (%)	
Bilateral	107(79.9%)
Unilateral	27 (20.1%)
Anastomotic site, n (%)	
Proximal	22 (16.4%)
Distal	112 (83.6%)

**Fig. 2:**
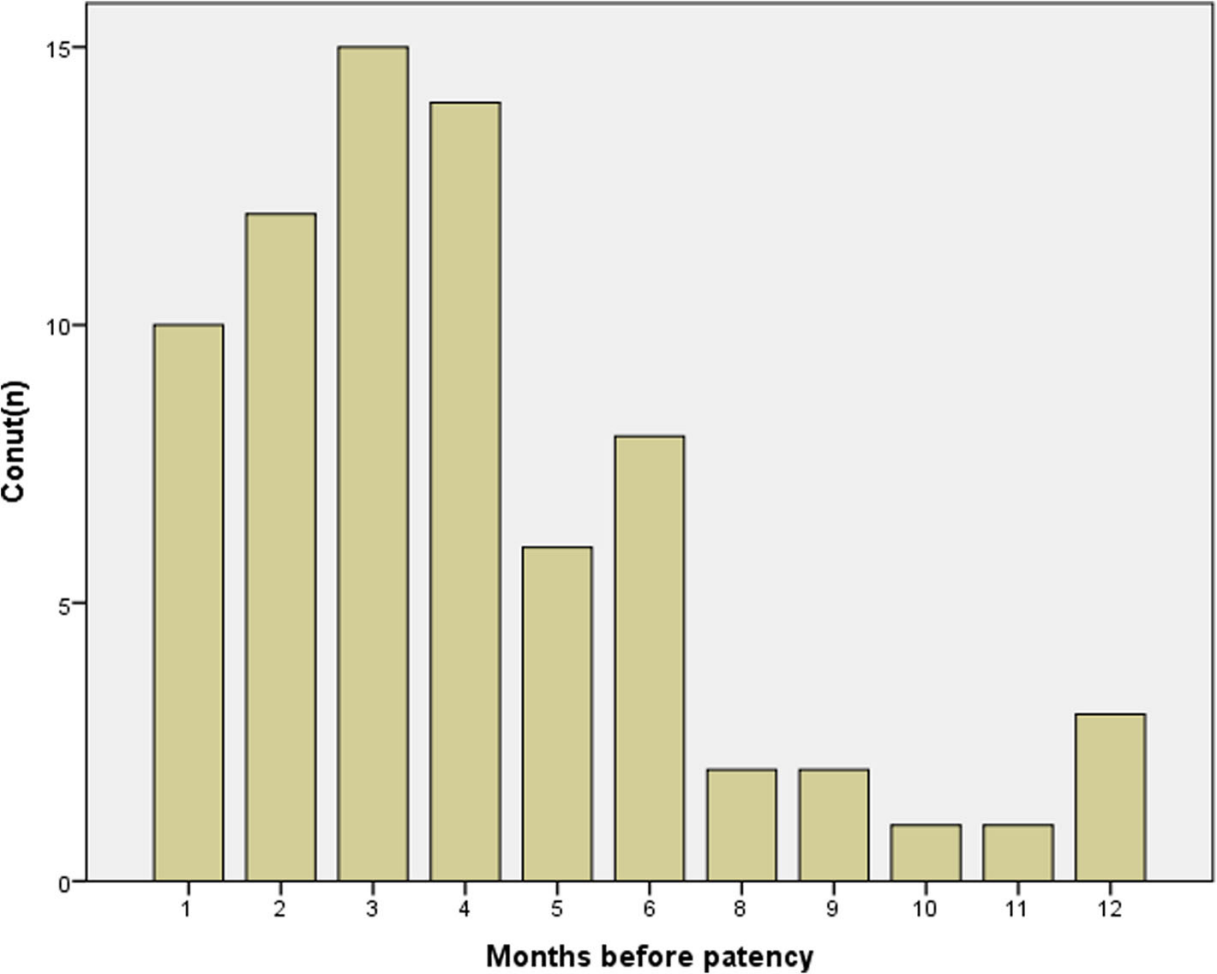
Time of sperm returning to the ejaculate. The column height of the histogram represents the patients counts within various periods to achieve patency

A total of 132 patients were subjected to sperm banking during the operation in the case of future intracytoplasmic sperm injection (ICSI). Sperm was present in the ejaculate of 74/134 (55.2%) patients after the surgery, and the average time to achieve patency was 4.11 ± 2.74 months. In the first 6 months post-treatment, 87.8% (65/74) patients were reported to have sperm in their ejaculate. Only 12.2% (9/74) patients claimed to achieve patency in the following 6 months (Fig. 2). The variables related to patency for follow-up are listed in Table 2. The patency rate was 58.9, 40.7, 36.4, and 58.9% for bilateral surgery, unilateral surgery, proximal anastomosis, and distal anastomosis, respectively. In this cohort, 30.3% (20/66) patients were reported to have spontaneous pregnancy after VE. The variables related to natural pregnancy are listed in Table 3. The natural pregnancy rate was 30.5% for bilateral surgery, 20% for unilateral surgery, and 33.3% for the site of distal anastomosis, but 0% for the proximal anastomosis. Furthermore, nine couples got pregnant by ICSI (seven used frozen sperm collected during the operation, and two used fresh sperm from the ejaculate). The total and natural pregnancy rate was 43.9% (29/66) and 30.3% (20/66), respectively, at the follow-up of 3–36 months. The mean time to achieve natural pregnancy was 11.05 ± 5.75 (range: 3–24) Months.

**Table 2:** The patency rate stratified by two variables for 134 patients

Variable	Patency (n)	*P* Value
Surgery		0.090
Bilateral	63/107 (58.9%)	
Unilateral	11/27 (40.7%)	
Anastomosis site		0.052
Proximal	8/22 (36.4%)	
Distal	66/112 (58.9%)

**Table 3:** The natural pregnancy rate stratified by two variables for 134 patients

Variable	Natural pregnancy (n)	*P* Value
Surgery		0.442
Bilateral	18/56 (32.1%)	
Unilateral	2/10 (20.0%)	
Anastomosis site		0.090
Proximal	0/6 (0%)	
Distal	20/60 (33.3%)	

## Discussion

Approximately 10–15% of infertile men suffer from azoospermia, while about 40% suffer from obstructive azoospermia (OA). This obstruction may be attributed to bilateral occlusion at any point in the reproductive ductal system, which comprises of the efferent duct, epididymis, vas deferens, and the ejaculatory ducts. Unlike the USA, EOA is rarely caused by vasectomy rather by infection in China [12, 13]. The microsurgical anastomosis, including microsurgical vasovasostomy (VV), cross vasovasostomy (CVV), and vasoepididymostomy (VE), is considered as the most successful measure for a reversal [14]. Herein, we discussed the modified single-armed 2-suture longitudinal intussusception vasoepididymostomy. The data were similar to the previously reported patency rate of 52–92% and the pregnancy rate of 11–56% [15, 16]. In the present cohort, we evaluated the pre-, intra-, and postoperative variables of individual patients that might affect the outcomes of the modified SA-LIVE.

The VE with the double-armed suture is the standard golden management for the EOA, and LIVE simplifies the anastomosis and improves the outcomes [7, 17]. However, in China, these specialized double-armed sutures for male infertility microsurgery are challenging, while access to single-armed microsurgical sutures is easy [18]. Furthermore, the cost of a single-armed sutures was cheaper than that of the double-armed microsurgical sutures. Therefore, SA-LIVE should be designated as an effective alternative when double-armed sutures are not available. When the needles are in the tubule, the two sutures in a lower position can avoid the crossing of the sutures [6]. The two knots of the suture are left outside during the procedure, which might decrease the possibility of fibrosis and anastomotic stricture. However, the single-armed suture is time-consuming as compared to the double-armed suture, which was placed inside-out on the vas deferens to avoid back-walling the tubular lumen. Since the single-armed suture was used in this study, the needle passed through the inferior points of the vasal mucosal layer in an outside-in manner through the epididymal tubule, and finally through the superior points of the vasal mucosal layer in an inside-out fashion, which was double time-consuming than the double-armed suture, thereby increasing the risks of surgery. Also, we had to dilate the vasal lumen sufficiently wide to pass the needle through the lumen with the aid of a microneedle holder to avoid back-walling during the suture placement. Supposedly, these procedures must be completed before the presence of the sperm in the epididymal fluid [19]. However, if no sperm was detected, the procedure was repeated 2–4 times, which would significantly increase the time required for the surgery and the fatigue of the surgeon.

In this cohort, the patency and the pregnancy rates were similar to those reported previously [12]. However, no statistically significant associations were found between the patency rate and various predictors, such as bilateral or unilateral anastomosis and anastomotic site (*P* > 0.05; Tables 2 and 3). Moreover, the natural pregnancy rate was 0% for the proximal anastomosis group, while it was 33.3% for the distal group, which prompted us to investigate whether patients can benefit from proximal anastomosis. Firstly, the luminal diameters of epididymal tubules in the caput were significantly smaller than those in the corpus and caudal, and hence, the modified SA-LIVE on the distal epididymis was more accessible than that on the proximal epididymis. Moreover, spermatozoa could become fully motile as well as recognize and fertilize an egg within the epididymis, which might improve the pregnancy rate. As the sample size was not sufficient for further investigation, the reason for the failure of the technique is yet to be determined.

Although there have been many clinical studies on VE, none proposed a specific follow-up time frame on SA-LIVE. The current study revealed that 87.8% of the patients achieved patency within 6 months post-operation, and none achieved patency since then, suggesting that SA-LIVE on patency can be followed up to 12 (average: 4.11 ± 2.74) months. The patency rate for the first 6 months was 48.5% (65/134) and 11.4% (9/69) for the next 6 months. Previous data on the mean time to achieve patency after vasovasostomy and vasoepididymostomy ranged from 1.7–4.3 and 2.8–6.6 months, respectively [12], which is in agreement with the current results. This finding could be valuable for the clinicians and researchers to predict the outcome of patients undergoing SA-LIVE as well as the precise time to transfer to ART.

Notably, four patients were diagnosed as non-obstructive azoospermia in other hospitals previously according to the negative results of the testis histopathology; however, we found motile sperm through the routine testicular biopsy. Thus, the scheduled operation of microdissection was transferred to VE, and 3/4 patients achieved patency afterward. This suggested that the testis biopsy during the operation is essential [20].

Also, one patient, who had undergone VE in another hospital previously was subjected to VE operation due to the previous failure. Consequently, sperm appeared in the ejaculate, and finally, this couple achieved a spontaneous pregnancy. Thus, we concluded that in addition to ICSI, a second VE surgery might be a possibility for those patients who failed the first operation.

Interestingly, VE is an effective treatment for azoospermia patients with epididymal obstruction and previous failure to achieve pregnancy by sperm retrieval with ICSI [21]. Three patients diagnosed with OA chose to use the sperm from testicular puncture for ICSI in another hospital but failed possibly due to miscarriage, maturation arrest in utero, or the failure of embryo transfer [21]. After undertaking SA-LIVE in our hospital, all the patients achieved patency: one had natural pregnancy, one underwent IVF after the surgery, and one is still trying to get a natural pregnancy. Furthermore, MEV has significant advantages, such as cost-efficiency, spontaneous pregnancy possibilities, and decreasing the potential risks of congenital disability as compared to ICSI. Thus, microsurgical reconstruction is an effective treatment and should be the first choice for epididymal obstruction patients whose female partners have normal fertility features. Thus, EOA patients with prior failure of ICSI could consider taking LIVE to get pregnant.

This study revealed that if sperm did not show up in the ejaculate after one-year post-operation, ART might be the remedy for such patients. Thus, intra- or postoperative sperm cryopreservation would be helpful. Intraoperative sperm cryopreservation could avoid the additional surgeries for sperm retrieval in case of failure of microsurgery. Postoperative sperm cryopreservation allows sperm from the ejaculate to be used for ART in the event of late failure. In the current cohort, we found out that one patient who achieved recanalization of the vas experienced a recurrence of obstruction after 6 months at the rate of 0.07%, which was lower than that reported previously (1–50%) [12, 22]. Sperm cryopreservation was used for a total of 132 patients. Of these, 29 patients who were not successful by vas-mediated patency achieved pregnancy through ICSI by the sperm cryopreserved intraoperatively, and nine successful patients chose to use the sperm from ejaculate to have babies through ART. The pregnancy rate was improved from 30.3 to 43.9%. With the development of the ART technique in the recent decades, the influence of whether the sperm on the next generation was unclear irrespective of its origin from testis or post-epididymis. Thus, taken together, we advocate intra- or postoperative sperm cryopreservation for all OA patients.

## Conclusion

Modified SA-LIVE is preferred when double-armed sutures are not accessible. The expected patency time is within one-year, and ART may be the remedial choice for patients. Assisted with intra-operative sperm cryopreservation, the pregnancy outcomes were improved in patients. The low natural pregnancy rate for the proximal anastomosis suggested that when the obstruction occurs proximally, sperm banking is preferred for future ICSI.

## Data Availability

The datasets used and analyzed during the current study are available from the corresponding author on reasonable request.

## Abbreviations

SA-LIVE: Modified single-armed 2-suture longitudinal intussusception vasoepididymostomy
VE: Microsurgical vasoepididymostomy
ART: Assisted reproductive techniques
FSH: Follicle-stimulating hormone
T: Testosterone
VV: Vasovasostomy
ICSI: Intracytoplasmic sperm injection
OA: Obstructive azoospermia
EOA: Epididymal obstructive azoospermia
IVF: In-vitro fertilization
